# Supplementary figures and images for: A microtubule RELION-based pipeline for cryo-EM image processing
Source: J Struct Biol. 2020 Jan 1;209(1):107402. doi: 10.1016/j.jsb.2019.10.004 (PMC6961209; doi:10.1016/j.jsb.2019.10.004)

**Manual picking**

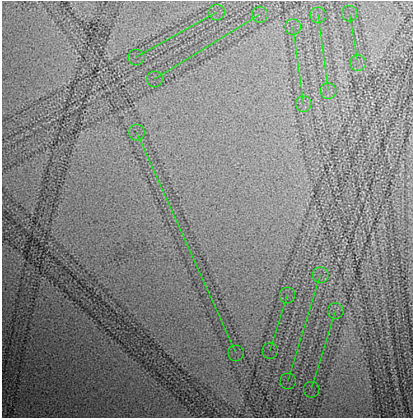

**Manual picked coordinates**

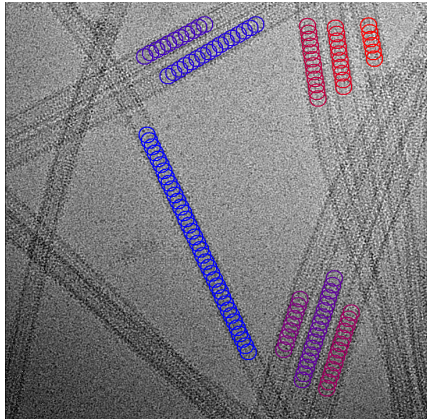

**Auto picked coordinates**

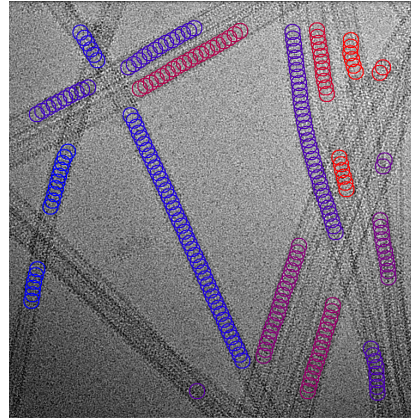

Supplement: Supplementary Fig. 1 — Methods of picking MT coordinates in RELION. In MiRP we pick MT coordinates manually in the manual picking RELION GUI by specifying roughly centred start and end coordinates for straight, non-overlapping stretches (left panel). From these start-end coordinates, unique segment coordinates are then specified using an inter-box distance matching the helical repeat distance upon extraction (central panel). In our hands autopicking in RELION generally produces suboptimal results (right panel), with some coordinates specified over curved MT regions and MT overlaps and many picked MT lengths being very short, thus providing poor statistical power for MiRP processing based on MT internal consistency. In central and right hand panels, MTs are coloured to illustrate different individual MT portions specified in RELION, based on RELION’s rlnHelicalTubeID parameter. [file mmc1.pdf]

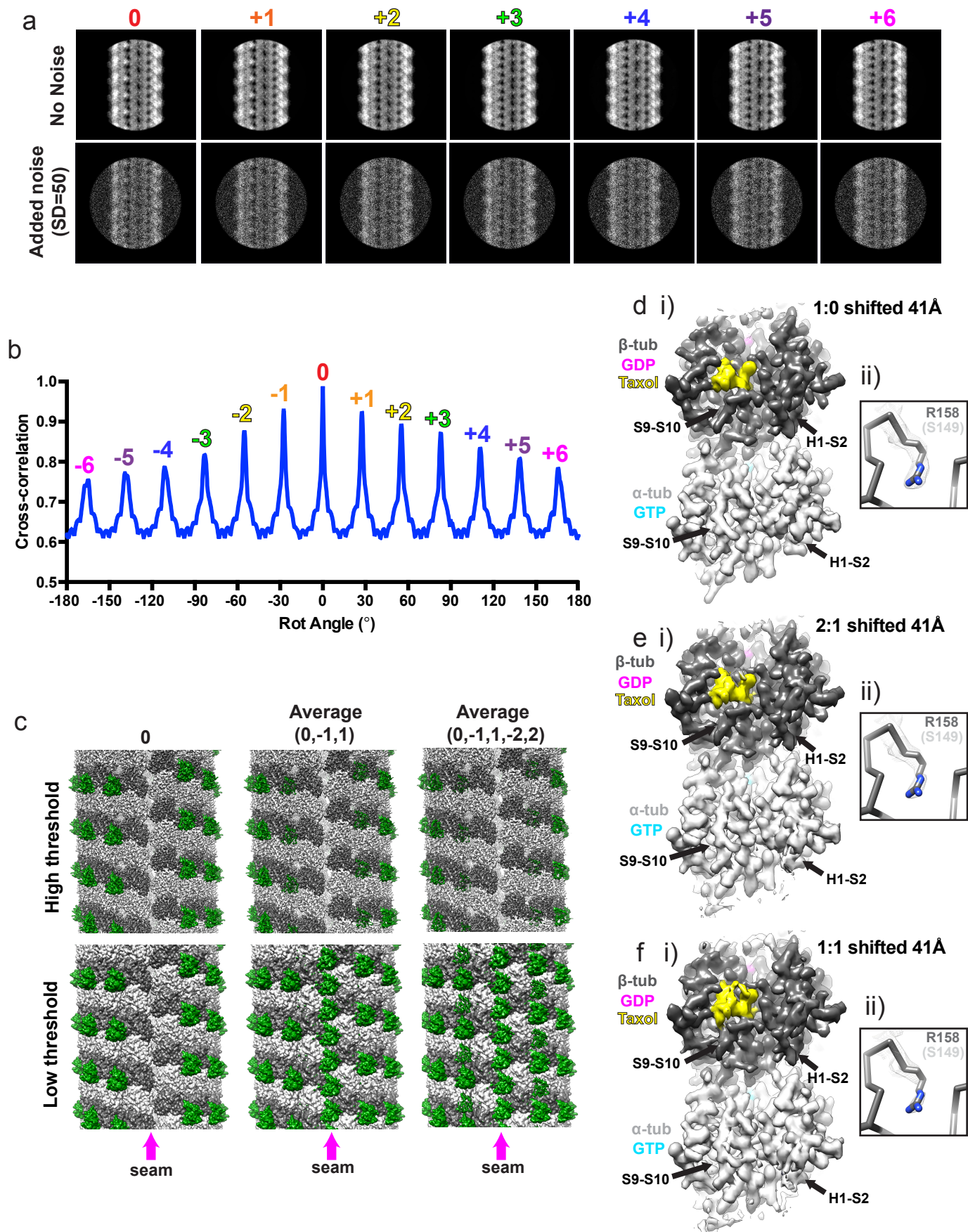

Supplement: Supplementary Fig. 2 — The challenges and pitfalls resulting from pseudosymmetry in MT processing. Each 2D projection of a given seam-containing MT has a number (according to the PF number) of highly similar but not identical pseudo-symmetry related projections designated by the helical twist and rise parameters, posing a unique challenge to processing MTs. a) 2D projections of a 13 PF CKK-decorated simulated reference, with (bottom row) or without (top row) simulated noise characteristic of low-dose cryo-EM images. Each 2D projection is related to the left-most panels (‘0’) by multiples of the helical twist and rise (+1 to +6), illustrating the similarity in pseudo-symmetry related images, which regularly results in alignment errors. The use of segment averages aims to increase the signal to noise ratio of MT 2D projection images. b) Cross-correlations between a given simulated 13PF CKK-MT 2D projection and its corresponding 3D 13PF CKK-MT reference in the absence of noise, where the highest cross-correlation results from the correct alignment ‘0’. As the alignment parameters are modified to represent rotation and translation along its helical path, cross-correlation peaks related by multiples of the helical twist and rise (-6 to +6) are apparent, with the higher peaks closest to the correct Rot angle and translation. c) The typical symptoms of poor MT rot angle determination on C1 reconstructions can be illustrated using a simulated (to 3.7 Å resolution) CKK-MT reference (left panels, ‘0’) averaged with two references rotated +1 or −1 multiples of the helical twist and rise (central panels, Average 0,−1,1) or these plus an additional two references rotated +2 or −2 multiples of the helical twist and rise (right panels, Average 0,−1,1, −2, 2). The 3D volumes are presented at high (top panels) and low (bottom panels) thresholds, with CKK density coloured in green. d-f) Effects of aberrant averaging of α- and β-tubulin density illustrated with tubulin density simulated to 3.7 Å resolution. [file mmc2.pdf]

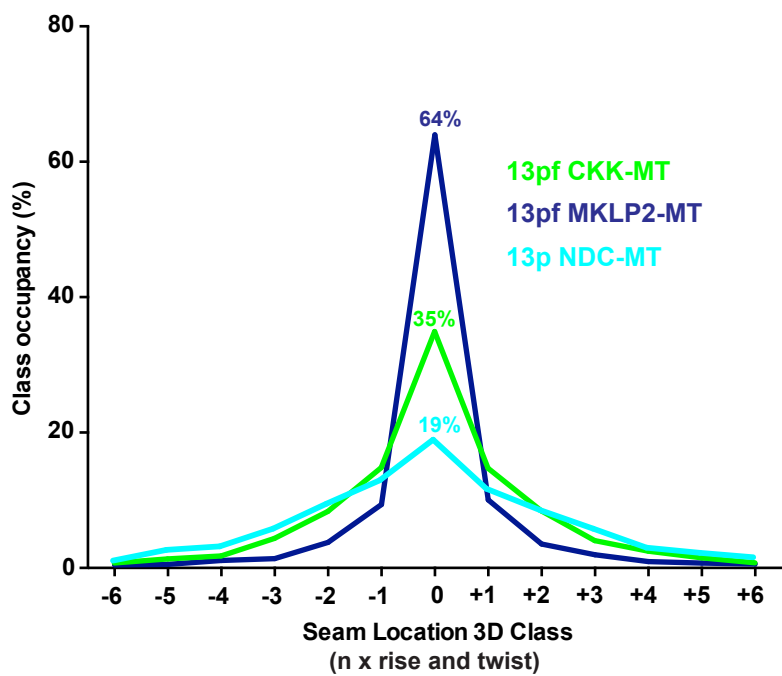

Supplement: Supplementary Fig. 3 — Seam finding 3D class allocation distribution for test datasets. Class occupancy distribution of 13 PF particles from CKK, MKLP2 and NDC decorated datasets classifying to 13 PF references built from appropriate decorating protein only density, with seams in modified positions (modified by -6 to +6 multiples of the helical rise and twist). [file mmc3.pdf]

MiRP

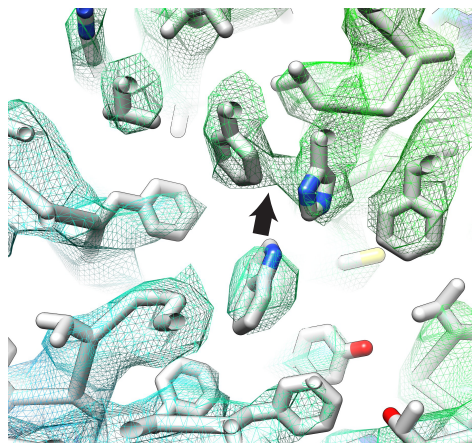

MiRP  
+ Bayesian polishing  
+ CTFrefine

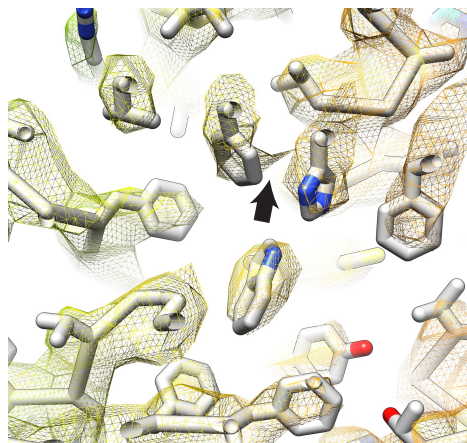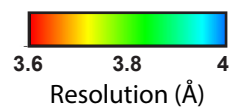

Supplement: Supplementary Fig. 5 — Resolution improvements after Bayesian polishing and CTF refinement. A central portion of α-tubulin model and density from the symmetrised CKK-decorated asymmetric unit (density shown as mesh), coloured by local resolution, is shown upon completion of the MiRP procedure at equivalent thresholds preceding or following single iterations of Bayesian polishing and CTF refinement procedures in Relion. [file mmc5.pdf]

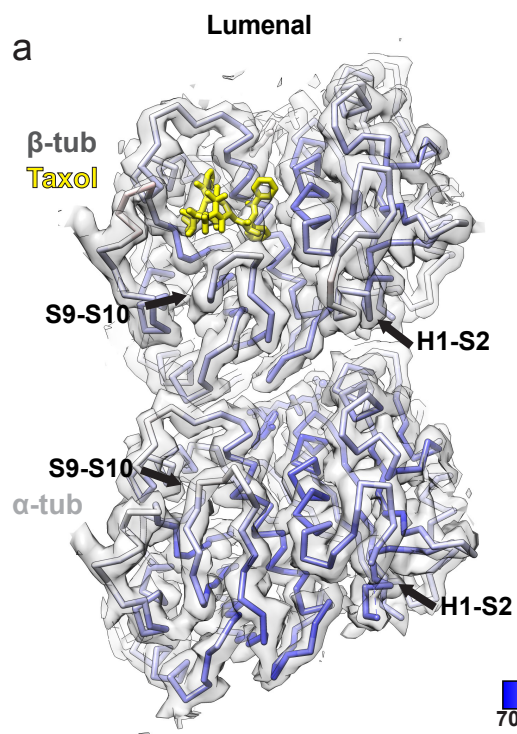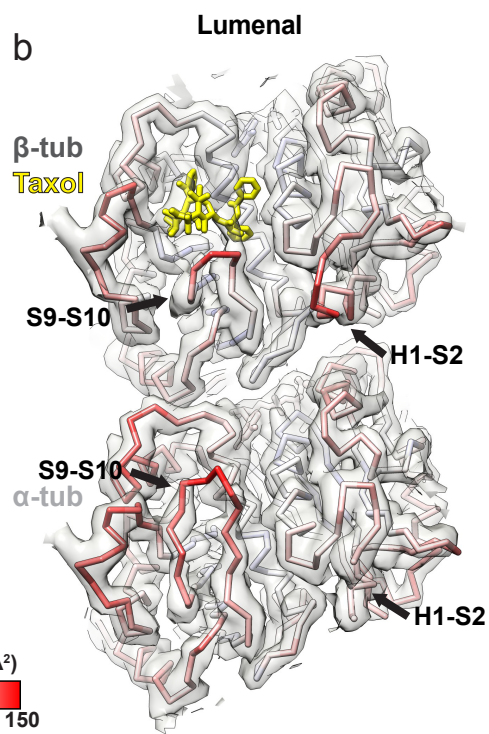

Supplement: Supplementary Fig. 6 — MiRP improves refinement B-factors in α- and β-tubulin. Refinement B-factors after PHENIX real-space refinement (Afonine et al., 2018) of the CKK-bound tubulin model into a) shown MiRP-derived density or b) shown standard helical processing-derived density (showing particularly high B-factors in structurally distinct regions between α- and β-tubulin such as the S9-S10 and H1-S2 loops). [file mmc6.pdf]

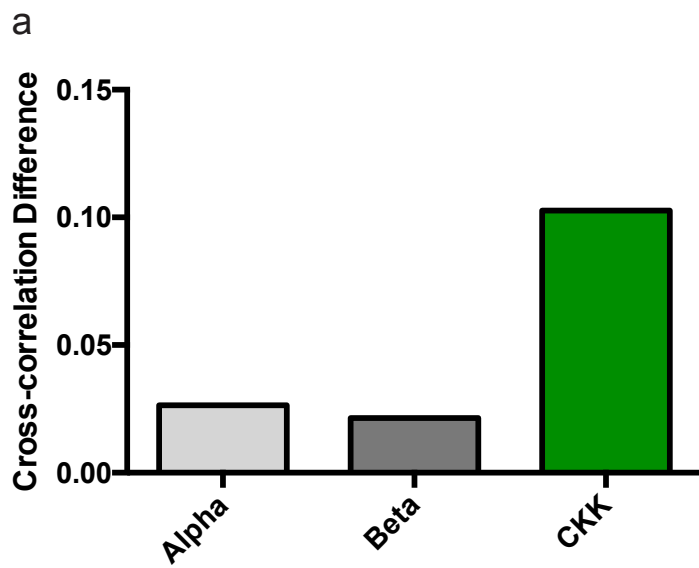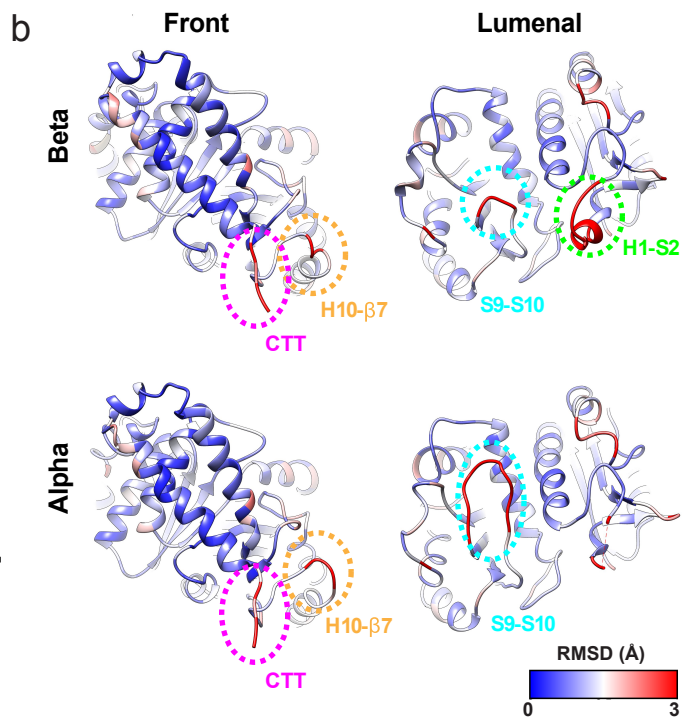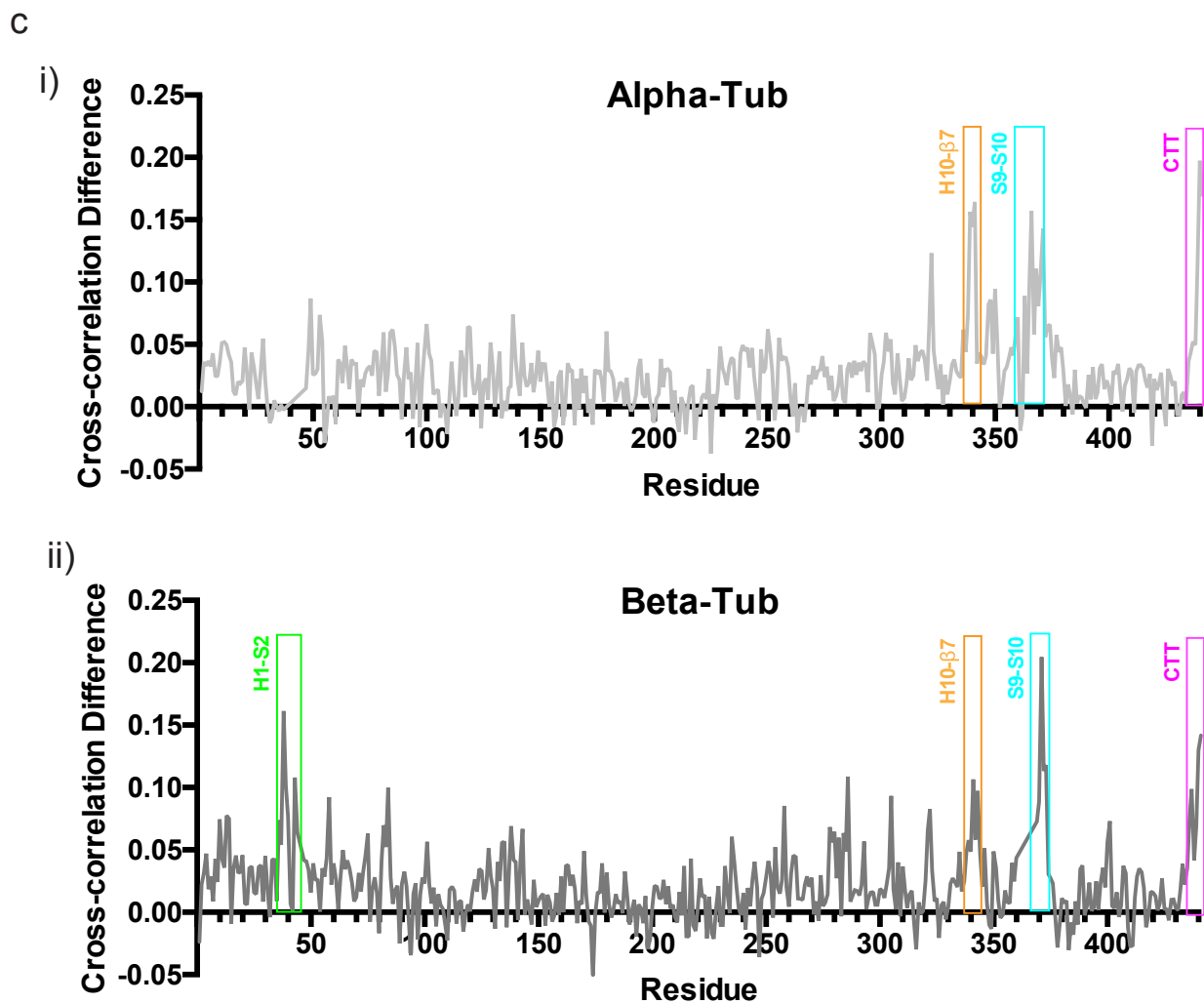

Supplement: Supplementary Fig. 7 — CKK-MT dataset map to model cross-correlations demonstrate that MiRP improves MT-Rot angle and αβ-tubulin register determination. a) Map to model local cross-correlations were calculated between the model built from MiRP-derived density (Atherton et al., 2019) and the CKK-decorated asymmetric unit density derived from either standard helical processing or the MiRP procedure. The former values were then subtracted from the latter to give cross-correlation differences. Asymmetric unit cross-correlation improves most for the CKK region of the reconstruction because it is most sensitive to the successful alignment performed by MiRP; global tubulin density and therefore model fitting is always good, even in reconstructions calculated using standard helical processing. b) Model RMSD between superimposed α- and β-tubulin models (MiRP-derived) shown on either front or lumenal faces of α- or β-tubulin. Structural regions of particularly high RMSD, indicating significant structural differences, are indicated with dashed rings. c) Per-residue differences in cross-correlation show local improvements from the MiRP procedure for i) α- and ii) β-tubulin, with improvements highest for indicated regions of high structural divergence corresponding to those identified in panel b. [file mmc7.pdf]

a

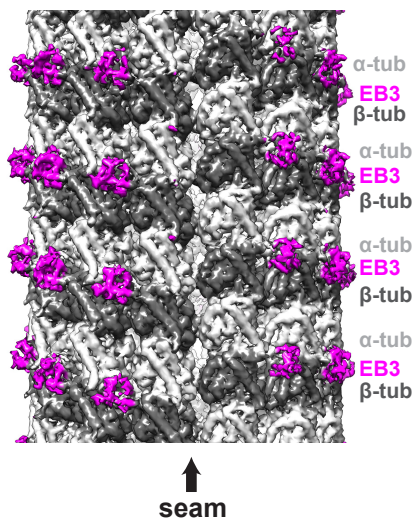

b

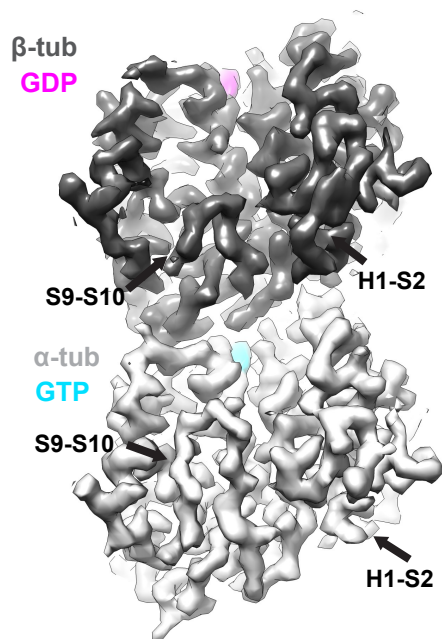

c

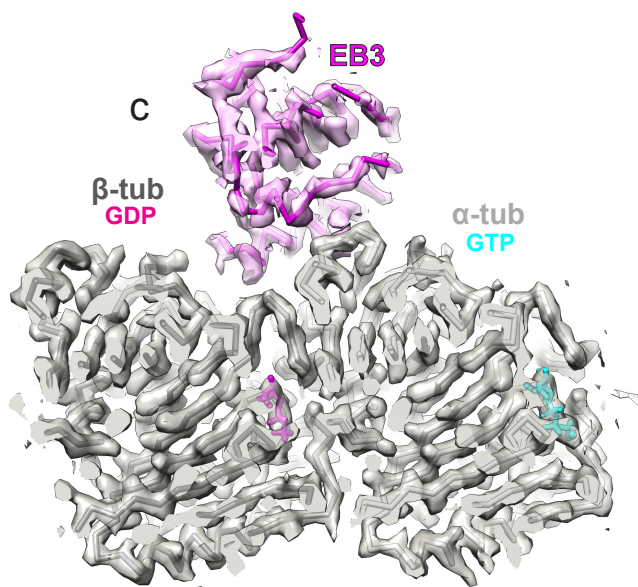

d

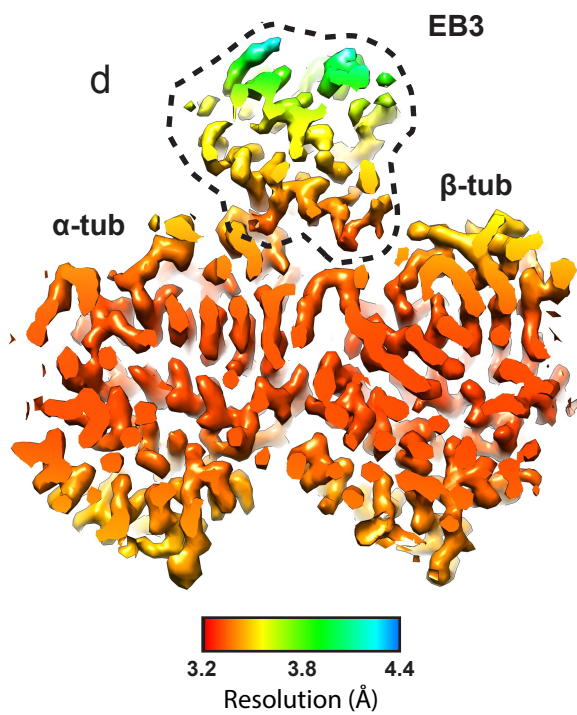

Supplement: Supplementary Fig. 8 — Results of MiRP processing of the publically available EB3-GDP-MT dataset (EMPIAR-10030). a) C1 reconstruction of the 13 PF EB3-GDP-MT dataset (unfiltered), showing a well-defined seam indicative of accurate MT Rot angle and αβ-tubulin register assignment. b) The lumenal face of the tubulin dimer of the asymmetric unit opposite the seam for symmetrised 13 PF EB3-MT reconstructions exhibits well defined density for the H1-S2 and S9-S10 loops, which are distinct in α- and β-tubulin. c) Density and fitted model for the ‘good’ asymmetric unit opposite the seam in the symmetrised reconstruction of the 13 PF EB3-MT dataset, showing density quality consistent with the reported resolution. d) As in panel c but from a different viewpoint, showing local resolution determined by RELION’s local resolution software. The EB3 decorating protein is within the dashed black line. [file mmc8.pdf]
