# Supplementary material for: A microtubule RELION-based pipeline for cryo-EM image processing
Source: J Struct Biol. 2020 Jan 1;209(1):107402. doi: 10.1016/j.jsb.2019.10.004 (PMC6961209; doi:10.1016/j.jsb.2019.10.004)
Supplement: Supplementary Fig. 4 — Testing the seam check 3D classification and MT-Rot angle/translational correction procedure. Unbinned datasets corresponding to MTs (particles after per-MT unification), classifying to CKK decorating protein only 3D references, with seams in modified positions (modified by −1 to 0 multiples of the helical rise and twist) shown in the first column were extracted. These datasets were subjected to multi-iteration local C1 refinements to new unbinned references, shown in column 2, of tubulin with decorating protein with a modified seam position. The 3D reconstructions from these refinements without (column 3) or with (column 4) correction of the MT Rot angles and translations are shown. [file mmc4.pdf]

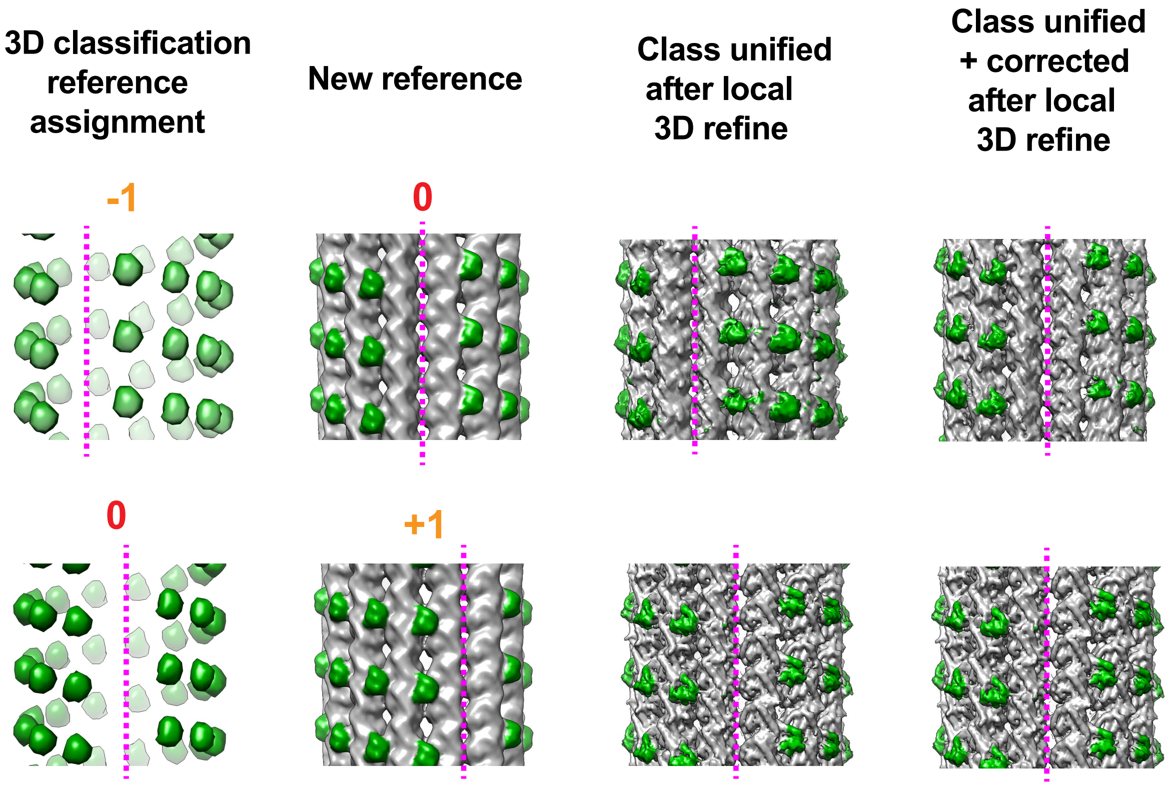
3D classification reference assignment
New reference
Class unified after local 3D refine
Class unified + corrected after local 3D refine
-1
0
0
+1
